# Allopatric integrations selectively change host transcriptomes, leading to varied expression efficiencies of exotic genes in *Myxococcus xanthus*

**DOI:** 10.1186/s12934-015-0294-5

**Published:** 2015-07-22

**Authors:** Li-Ping Zhu, Xin-Jing Yue, Kui Han, Zhi-Feng Li, Lian-Shuai Zheng, Xiu-Nan Yi, Hai-Long Wang, You-Ming Zhang, Yue-Zhong Li

**Affiliations:** State Key Laboratory of Microbial Technology, School of Life Science, Shandong University, Jinan, 250100 China

**Keywords:** Transposition, Site-directed integration, Chloramphenicol acetyl transferase, Epothilone biosynthetic gene cluster, Expression efficiency, Transcriptome, *Myxococcus xanthus*

## Abstract

**Background:**

Exotic genes, especially clustered multiple-genes for a complex pathway, are normally integrated into chromosome for heterologous expression. The influences of insertion sites on heterologous expression and allotropic expressions of exotic genes on host remain mostly unclear.

**Results:**

We compared the integration and expression efficiencies of single and multiple exotic genes that were inserted into *Myxococcus xanthus* genome by transposition and *attB*-site-directed recombination. While the site-directed integration had a rather stable chloramphenicol acetyl transferase (CAT) activity, the transposition produced varied CAT enzyme activities. We attempted to integrate the 56-kb gene cluster for the biosynthesis of antitumor polyketides epothilones into *M. xanthus* genome by site-direction but failed, which was determined to be due to the insertion size limitation at the *attB* site. The transposition technique produced many recombinants with varied production capabilities of epothilones, which, however, were not paralleled to the transcriptional characteristics of the local sites where the genes were integrated. Comparative transcriptomics analysis demonstrated that the allopatric integrations caused selective changes of host transcriptomes, leading to varied expressions of epothilone genes in different mutants.

**Conclusions:**

With the increase of insertion fragment size, transposition is a more practicable integration method for the expression of exotic genes. Allopatric integrations selectively change host transcriptomes, which lead to varied expression efficiencies of exotic genes.

**Electronic supplementary material:**

The online version of this article (doi:10.1186/s12934-015-0294-5) contains supplementary material, which is available to authorized users.

## Background

Heterologous expression is a routine laboratory technique to obtain massive quantities of interesting products, not only single recombinant proteins but also metabolites from complex biosynthetic pathways, such as microbial secondary metabolites. Efficient expression and high yields of exotic genes in acclimatized hosts are not only important for increasing the yields of desired products, but also useful in the discovery of novel compounds, for example, the products of those cryptic secondary metabolic pathways [1, 2]. Heterologous expressions of single gene products can normally be achieved in high efficiency and yields in *Escherichia coli* after codon optimization, promoter selection, co-expression with chaperon protein genes, and/or cultivation improvement [1–3]. However, heterologous expressions of metabolites from pathways containing cluster-organized multiple-genes confront much more difficulties and limits [4]. Researchers have to establish suitable transfer systems for large-sized gene clusters, maintain integrity of well-organized genes during transfer, trigger efficient expressions of multiple genes, provide sufficient supplies of substrates for the biosynthesis, facilitate secretion to eliminate products-feedback inhibition on biosynthesis or toxic effects on host, and reduce negative effects of local metabolisms on the desired expressions. Up to now, efficient expression of a complicated biosynthetic pathway in heterologous host is still a challenge.

Because of large size, multiple genes of complicated biosynthetic pathways are often integrated into chromosome for heterologous expression. For example, antitumor compounds epothilones were originally isolated from myxobacterial *Sorangium cellulosum* cultures [5]. The 56-kb biosynthetic gene cluster has been successfully introduced into different hosts, including *Streptomyces coelicolor* [6, 7], *S. venezuelae* [8], *E. coli* [9], *Pseudomonas putida* [10] and other myxobacterium *Myxococcus xanthus* [10–12]. While the yields of epothilones in *M. xanthus* might be up to 160 μg/L-titer [11], the yields in those distantly related hosts were lower than 1 μg/L, or undetectable [9, 10], probably due to incompatibility of the exotic DNA and/or metabolites. Thus, although distantly related hosts may have merits in genetic performances, growth and fermentation, closely related species turn to be preferable for heterologous expression of the products from large gene clusters. However, the productions of epothilones are also greatly varied in those engineered *Myxococcus* strains, even with almost identical genetic backgrounds [10, 11], suggesting internal uncertainty for the expression of multiple exotic genes. For example, it is yet unclear whether and how insertion sites influence the expression efficiency of exotic genes and whether and how allotropic expressions influence on host cells.

Transposition is able to bring exotic genes into host chromosome randomly. Recently, transposon technique has been developed for heterologous expression of large-sized gene clusters [10], which provides an approach for analysis of the influences of integration patterns. In this study, we compared expression efficiencies of the chloramphenicol acetyl transferase gene that was introduced into chromosome via transposition insertion or site-specific insertion. We then constructed vectors containing the entire 56-kb epothilone gene cluster, stitched using a modified recombination strategy, for one-step introduction into *M. xanthus* cells. We assayed effects of integration sites on the yields of epothilones and transcriptome changes in different mutants. Our results indicated that allopatric integrations selectively change host transcriptomes, leading to varied expression efficiencies of exotic genes in *M. xanthus*.

## Results and discussion

### Site-specific and transposition insertions of *cat* gene in *M. xanthus*

There are usually two ways to introduce exotic genes into *M. xanthus* genome. One is arbitrary insertion via transposon such as *miniHimar1*, a plasmid derived from the mariner element *Himar1* [13, 14], while the other is site-specific recombination, normally at the chromosomal *attB* site via *Mx8 att*, developed from myxophage *Mx8* DNA [15, 16]. To test whether insertion pattern influences heterologous expression, we introduced the chloramphenicol acetyl transferase (*cat*) gene into the genome of *M. xanthus* DZ2 via either transposition (Tp) or site-directed insertion (Mx8). Previous studies related to heterologous expressions of the epothilone biosynthetic gene cluster normally employed an exotic promoter like *aphII*, the kanamycin promoter in pKK-aphII [17], which is frequently used in *M. xanthus* [18, 19]. We once reported that the 843-bp *epoP* promoter for the biosynthetic gene cluster of epothilones from *S. cellulosum* So0157-2 exhibited much higher activities than *aphII* in *E. coli* [17]. Thus, four plasmids containing the respective promoter and integration elements, i.e. pTp-epoP, pMx8-epoP, pTp-aph and pMx8-aph, were constructed (Additional file 1: Figure S1), which were further electroporated into the *M. xanthus* DZ2 strain, respectively.

**Figure 1:**
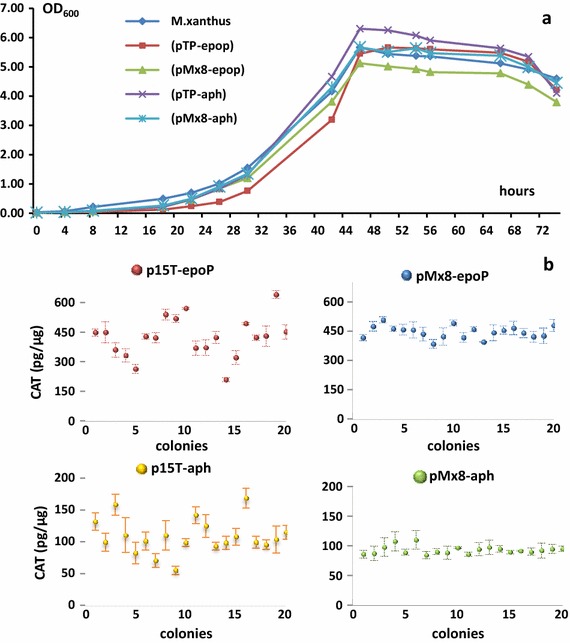
Growth and CAT activities of *M. xanthus* transformants integrated from the plasmids pTP-epoP, pMx8-epop, pTP-aph or pMx8-aph. **a** The growth curves of *M. xanthus* transformants, compared with *M. xanthus* DZ2. **b** The CAT expression activities in *M. xanthus* transformants. Twenty clones were selected randomly from each of the four recombinant categories.

Compared with the wild type strain, the *cat* expression had small effects on the growth of recombinant cells (Figure 1a). We picked-up 20 colonies randomly from each of the four recombinant categories to assay their chloramphenicol acetylation activities. The site-directed recombination produced rather stable CAT activities, i.e. approximately 450 and 100 pg/μg in the pMx8-epoP and pMx8-aph transformants, respectively. However, the pTp-epoP and pTp-aph strains showed highly varied CAT activities, ranging from 209.31 to 638.49 pg/μg in the pTp-epoP transformants or from 55.59 to 168.24 pg/μg in the pTp-aph transformants (Figure 1b). Furthermore, it is clear to see that the *M. xanthus* transformants from the pTp-epoP or pMx8-epoP plasmids exhibited several times higher CAT activities than those from the corresponding pTp-aph or pMx8-aph plasmids. The *epoP* promoter is also more efficient than the *aphII* promoter in *M. xanthus*. Accordingly, the original *epoP* promoter was used for further heterologous expressions of the epothilone biosynthetic genes.

### Construction of plasmids containing the entire epothilone biosynthetic gene cluster

Epothilones are biosynthesized by a gene cluster of seven multifunctional modules, spanning approximately 56 kb in length [20, 21]. In this study, we constructed the whole epothilone biosynthetic genes into single plasmids for the integration in *M. xanthus* chromosome. The epothilone genes were from the Cosmid10 and Fosmid3B11 plasmids, which separately contained a 38.5-kb fragment from *epoA* to the front part of *epoD* and a 34.4-kb fragment from the rear part of *epoC* to the downstream fragment of *epoF*. The two fragments overlapped a 6.5-kb region.

**Figure 2:**
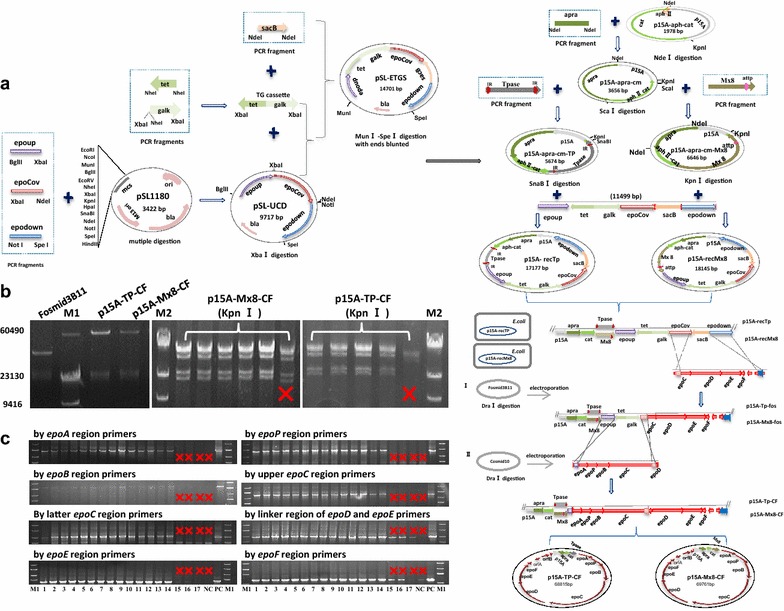
Construction of plasmids containing the epothilone biosynthetic gene cluster and their expressions in *M. xanthus*. **a** Diagram of the plasmid construction. The plasmid p15A-recTp and p15A-recMx8 contain the transposase gene *(IR*-*Tpase*-*IR*) and the site specific attachment element (*Mx8*), respectively. Homologous arms (*epoup*, *epoCov*, *epodown*) and selective genes *(tet*, *apra*, *galk*, *sacB*) were constructed into the plasmids. The construction of the plasmids p15A-TP-CF and p15A-Mx8-CF, carrying the entire epothilone gene cluster, were performed by two rounds of recombination. Firstly, the linearized Fosmid3B11 was electroporated into the *E. coli*, which recombined with p15A-recTp or p15A-recMx8 to generate the plasmid p15A-Tp-fos or p15A-Mx8-fos. Then, the linearized Cosmid10 was introduced for recombination with p15A-Tp-fos or p15A-Mx8-fos to create the p15A-TP-CF or p15A-Mx8-CF. **b** PCR amplification verification of constructs. *Left panel* shows the size indication of the plasmids p15A-TP-CF, p15A-Mx8-CF and Fosmid3B11. The *middle* and *right panels* are digestion analyses of p15A-Mx8-CF and p15A-TP-CF colonies by restriction enzymes KpnI, respectively. M1 and M2 are DNA markers. **c** PCR amplification verification of the presence of epothilone biosynthetic genes in *M. xanthus* recombinants. The *cross marks* in **b** and **c** indicate the false positive clones.

To simplify recombination and to avoid false-junction, we stitched the two fragments into a complete epothilone gene cluster using a one-stop-way strategy. The homologous arms included an upstream arm, a downstream arm and a shared middle arm. The shared middle arm was designed to stitch the two large fragments, permitting the two rounds of recombination happening in no particular order for integration of the epothilone gene cluster fragments into plasmid. The three homologous arms were constructed into a single vector, and the stitching was carried out in *E. coli* GB05-red by means of the red-ET recombination system (diagrammed in Figure 2a). The correct colonies were screened by the negative marker *galk* and *sacB*, and the junction regions were verified by restriction digestion (Figure 2b). After sequencing verification, we got the correct recombinant plasmid p15A-Tp-CF (68.8 kb).

We also constructed a recombinant plasmid containing the *Mx8 att* sequence (Figure 2a, b) for site-specific recombination.

### Integration of the epothilone biosynthetic genes into *M. xanthus* chromosome

The plasmids were subject to one-step integration into *M. xanthus* DZ2 chromosome, and the recombinant colonies were screened by selection markers Apra and Cm. After 6–8 days of incubation at 30°C, we obtained 21 colonies from the CYE plate plus the Apra and Cm antibiotics in a single transformation performance. PCR amplification determined that 14 of the 21 antibiotics-resistant colonies were correct transformants, while the other seven contained partial or none of epothilone genes. A further transformation performance of p15A-Tp-CF in *M. xanthus* DK1622 strain yielded 30 correct transformants from 59 antibiotics-resistant colonies. We also performed transformations in SW504, a *difA* mutant of *M. xanthus* DK1622 [22, 23], from which 4 correct transformants were obtained. Figure 2c demonstrates some PCR amplification results. Our results showed that the transposition technique was highly efficient to nail large exotic genetic fragments into the chromosome of different *M. xanthus* strains.

Electroporation of the p15A-Mx8-CF plasmid with *M. xanthus* DZ2 strain obtained six tiny colonies on the selection plate, which, however, were turned out to be spontaneous mutants without any epothilone genes. We performed transformation of the plasmid in different *M. xanthus* strains, which also failed in obtaining any correct transformant. To testify whether the failure was due to the size limitation for integration at the *Mx8 att* site, we constructed smaller plasmids of 42.0-kb p15A-Mx8-fos, derived from the recombination of the linear fosmid3B11 with p15A-recMx8, and 18.1-kb p15A-recMx8. The transformation performance with these two plasmids under the same conditions produced 4 and 47 correct colonies in *M. xanthus* DZ2, respectively. We thus concluded that the recombination efficiency of the *Mx8 att* site decreased sharply with the increase of the integration fragment sizes.

### Epothilone production abilities and integration sites in the transposition transformants

**Figure 3:**
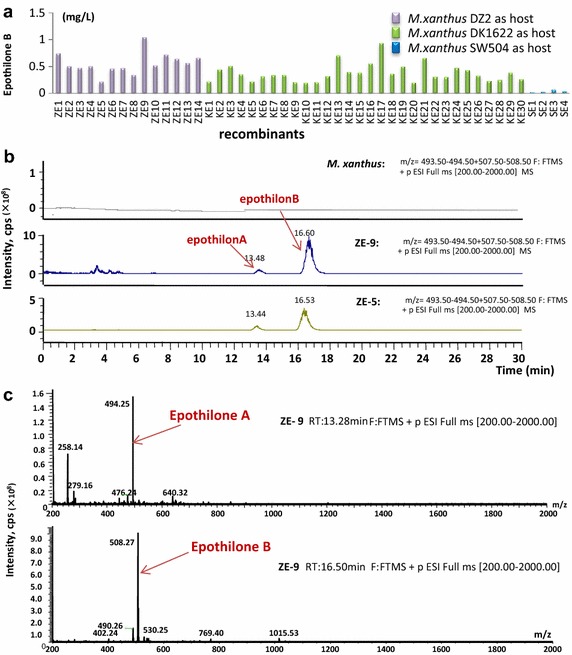
Production of epothilone B in the *M. xanthus* transformants (**a**) and HPLC–MS detection (**b**, **c**). **a** The epothilone productions, assayed after 7-days fermentation. ZE-, KE- and SE- series strains are termed for those recombinants from *M. xanthus* DZ2, DK1622 and SW504 strains, respectively. **b** HPLC results of the ion chromatograms of HPLC–MS runs. The *upper row*, a negative control form the wild type *M. xanthus* DZ2. The *middle* and the *lower rows* demonstrate the results from ZE-5 and ZE-9, respectively. **c** The fragmentation patterns of epothilone A and epothilone B produced in the isolate no. 9 with the correct molecular weight minus the H^+^ (epothilone A = 493; epothilone B = 507).

We assayed the production abilities of epothilones in 48 correct recombinant transformants derived from different *M. xanthus* strains, i.e. DZ2, DK1622 or SW504. The strains were cultivated in the CYE medium for 5 days of incubation, and the production of epothilones was characterized by high performance liquid chromatography-mass spectrometry (HPLC–MS) techniques. The results showed that all these recombinants were able to produce epothilones A and B; whereas no epothilone was detectable in the original *M. xanthus* strains (Figure 3a; detailed information of the yields in different recombinants is provided in Additional file 2: Table S1; Figure 3b, c demonstrate the HPLC–MS detection of epothilones production). Similar to that of the CAT enzyme activities in transposition transformants, the yields of epothilones varied significantly in these recombinants. For example, in the *M. xanthus* DK1622 host, the yields in CYE medium ranged from 0.045 to 0.17 mg/L of epothilone A and 0.22–1.05 mg/L of epothilone B. Among the three employed *M. xanthus* hosts, the yield ranges were similar in the DZ2 and DK1622 mutants, but much low in the SW504 mutants. The production abilities of epothilones in *M. xanthus* transposition recombinants from DZ2 or DK1622 were rather similar to that in those wild type epothilone-producing *S. cellulosum* strains [24]. However, in contrast to the 2:1 ratio of epothilones A to B or almost single A without B in *S. cellulosum* strains [24], the ratios of epothilones A–B produced in these *M. xanthus* producers were all approximately 1:5 (Additional file 2: Table S1). Compared to those previously reported epothilone-producing *M. xanthus* recombinants (approximately 0.1 mg/L or less) [10, 11], some of the transposition transformants gave much higher productions under unimproved fermentation conditions.

**Figure 4:**
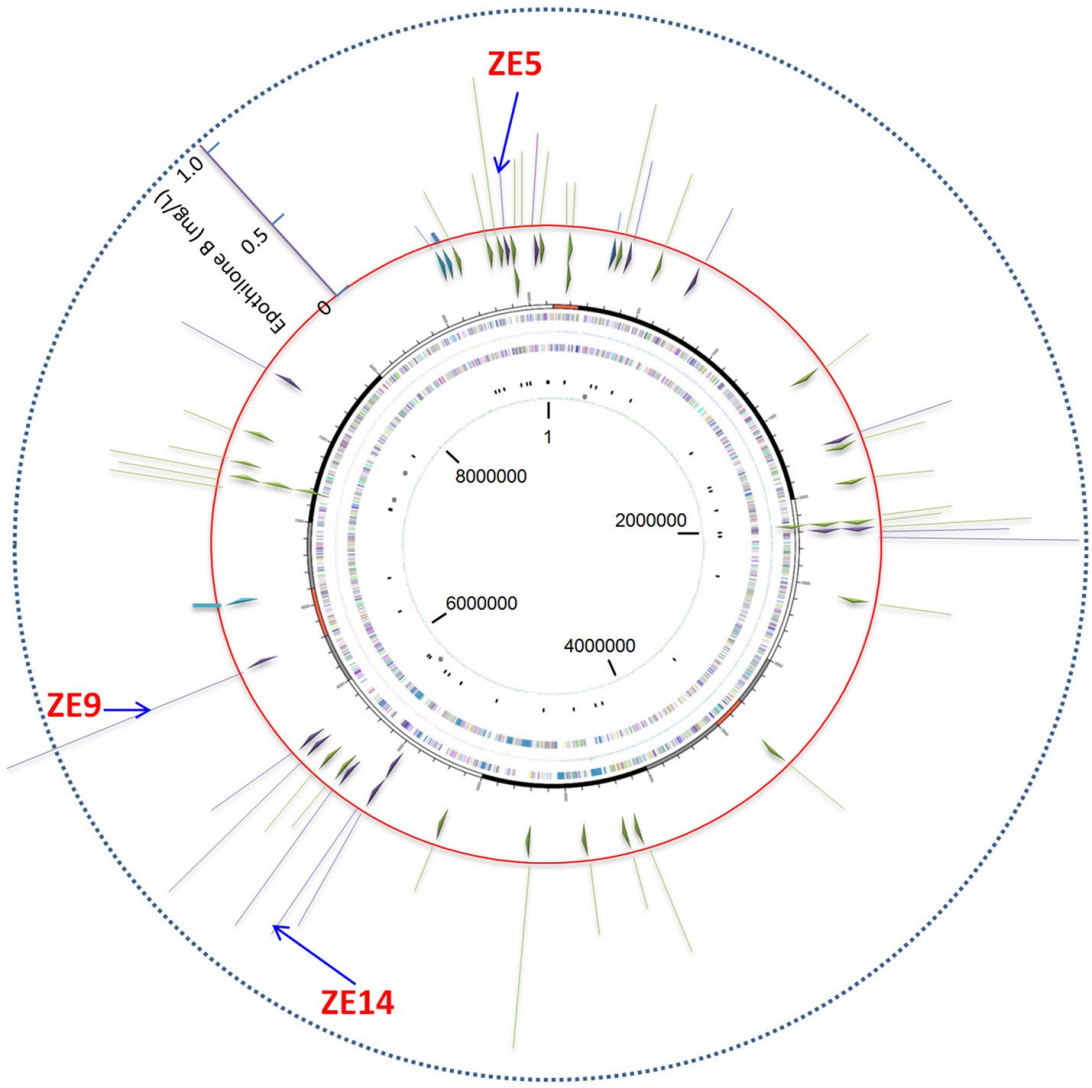
Distribution of the integration sites of the epothilone genes (the *small triangles* inside the *red ring*; all inserted clockwise) and the production abilities (the *small bars outside* the *red ring*) in 48 epothilone producing recombinants, mapping on the host chromosome. The *purple*, *green* and *blue bars* stand for recombinants derived from DZ2, DK1622, and SW504, respectively.

We mapped the integration sites of the epothilone genes in genome using the genome walking technique. Sequencing revealed that the epothilone genes were located at 41 different sites in the 48 recombinants, of which six were repeatedly inserted (Figure 4; details are provided in Additional file 2: Table S1). The epothilone genes were all in the same direction, and these integration sites scattered along the *Myxococcus* chromosome, seemly with no bias. The recombinants with the same integration sites also had similar yields of epothilones, which was consistent with that of the site-directed insertion of CAT gene at the *attB* site (Figure 1b). However, no clear correlation was observed between the production abilities of epothilones and the insertion sites in genome (Figure 4).

### Transcriptomics variation in epothilone-producing transposition transformants

**Figure 5:**
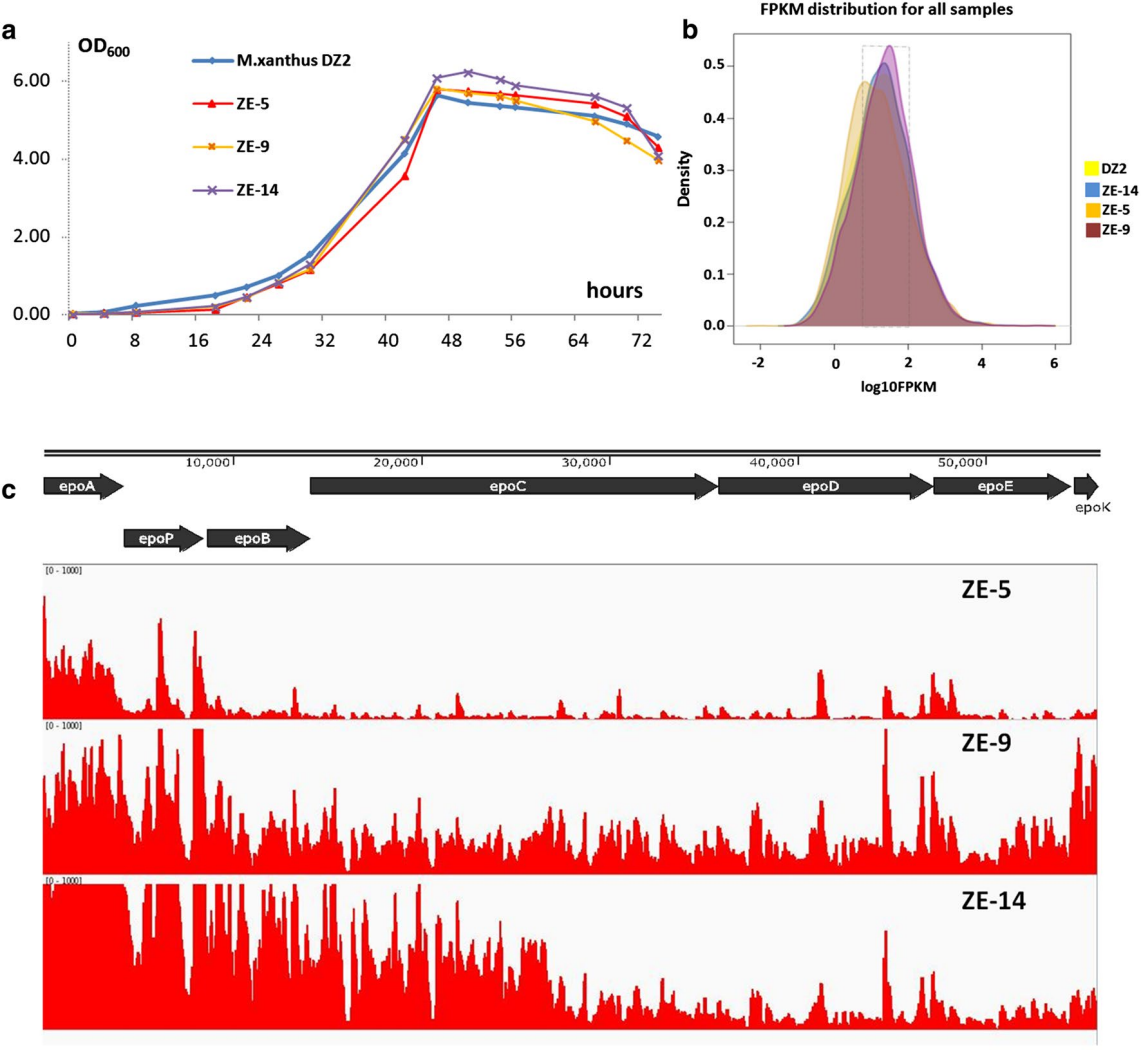
Transcriptomes of *M. xanthus* DZ2 and three recombinants with insertions of epothilone biosynthetic genes. **a** The growth curves of the ZE-5, ZE-9 and ZE-14 three recombinants, compared with DZ2. **b** Distribution of expression density (fragments per kilobase of expressions per million mapped reads, FPKM) of the total genes in the four strains. **c** Transcriptomics analysis of the expression of the epothilone genes.

The above results suggested that the production differences probably resulted from the influences of insertion sites on the expressions of the epothilone genes. In order to investigate how allopatric integrations influenced the yields of epothilones, we assayed the transcriptomes in three recombinants, as well as wild type strain DZ2. The three recombinant strains, *i.e.* ZE-5 (inserted at MXAN_7320), ZE-9 (inserted at MXAN_5011), and ZE-14 (inserted at MXAN_4403), were varied of their yields of epothilones in the CYE medium (0.22 ± 0.08, 1.05 ± 0.21 and 0.66 ± 0.09 mg/L epothilone B, respectively; Additional file 2: Table S1). To confirm the total biosynthetic genes were at the same place in each strain, we performed PCR amplification of the junction regions between the seven modules of the gene cluster. The results showed that all the modules were neighbored in each of these three recombinants (Additional file 3: Figure S2). Growth assays indicated that the three strains had similar growth curves as the wild type strain (Figure 5a). The RNA materials were extracted from the cells, which were collected when XAD-16 resin was harvested for the assay of epothilone production. After confirmation of the extracts free of DNA, we sequenced transcriptomes using strand specific RNA-seq technique (Illumina Miseq 2000 technique). The sequencing obtained 18.3, 21.3, 18.5 and 20.8 million reads for DZ2, ZE5, ZE9 and ZE14, respectively. Totally, 97.4–98.2% of reads were mapped to genome, and the distribution had good correlation with the gene number in each strand. We checked the expression levels of the insertion-mutated genes in the wild type strain DZ2 (Additional file 2: Table S1), which, however, showed no correlations with the production abilities in the transposition recombinants.

Comparative transcriptomes showed similar distributions of expression density of the total genes in the four recombinants (Figure 5b). The expression patterns of the epothilone genes varied significantly in the three recombinants (Figure 5c), but parallel to their production abilities. The expression levels of the epothilone biosynthetic genes in ZE-5 were low, especially the latter part of this big gene cluster. In contrast, the epothilone genes expressions in ZE-9, which had approximately five times of the epothilone production in ZE-5, were rather high in different regions in the gene cluster. In the ZE-14 strain, although expressions of the front region were much high, the hinder genes had sharply decreased expressions, which probably led to the medium level of the epothilone production.

Compared to the *M. xanthus* DZ2 strain, many *Myxococcus*-own genes were up- or down-regulated distinctly in these allopatric insertion mutants (Additional file 4: Table S2). For example, three hypothetical protein genes (MXAN_5126, MXAN_5530 and MXAN_3967) were up regulated, while two genes (MXAN_4480 predicted for a DNA binding protein and MXAN_4324 for a hypothetical protein) were down regulated in the ZE-9 and ZE-14 strains. Six genes were specifically down regulated of their expressions in the ZE-9 strain, but were not significantly changed in ZE-5 or ZE-14. In all the three tested recombinant strains, three *Myxococcus* genes, i.e. MXAN_1576 (predicted encoding a major facilitator family transporter), MXAN_1093 (a DNA-binding response regulator) and MXAN_7163 (bis (5′-nucleosyl)-tetraphosphatase, symmetrical), were found to be significantly up-regulated, while two genes, i.e. MXAN_7372 (hypothetical protein) and MXAN_4372 (DNA-binding protein) were significantly down regulated. It is noted that the locations of these up- or down- regulated genes normally had no relationship with the insertion sites in *Myxococcus* genome. These transcriptome changes indicated that allopatric integration of exotic genes in genome had some specific effects on the transcriptome in host, which probably resulted in distinct expressions of the exotic genes.

## Conclusions and outlook

In contrary to potential size-limitation of the inserted fragments at directed sites, demonstrated using the *attB* site in *M. xanthus*, transposition attempts to position large-sized genetic materials randomly into genome, thus provides much more opportunities for the integration of exotic genes. Such random transposition integration is blind, and the introduced exotic genes may inactivate or disturb functions of host genes, but also yields varied expression levels of exotic genes. The expression differences of allopatrically integrated genes did not follow the local expression characteristics, but rather the results of some global changes of the host transcriptome. We might be able to improve the expressions of the exotic genes further by genome engineering based on the transcriptome changes. In addition, the expressions of epothilone genes were rather different in different cluster regions–increasing the expression of low-expressed genes will be also useful for the yield improvement.

## Methods

### Bacterial strains, plasmids and culture conditions

Bacterial strains and plasmids used in this study are listed in Table 1. *Escherichia coli* DH5α was used for routine transformations and sub-cloning. *E. coli* GB05-red, a derivative of DH10B [25], was used in recombination performance. *E. coli* strains were grown routinely in Luria Broth (LB) medium (10 g/L peptone, 10 g/L yeast extract and 5 g/L NaCl; pH7.2), and *Myxococcus xanthus* strains were grown in CYE medium [10 g/L casitone, 5 g/L yeast extract, 10 mM 3-(N-morpholino) propanesulfonic acid (MOPS; pH 7.6) and 4 mM MgSO4] [26]. The growth temperatures were 37°C for *E. coli* and 30°C for *M. xanthus*, respectively. For selection of constructed plasmids or transformants, different antibiotics, i.e. ampicillin [Amp], 100 μg/mL; kanamycin [Km], 40 μg/mL; chloramphenicol [Cm], 10 μg/mL; or apramycin [Apra], 25 μg/mL, were added in LB or CYE media.

**Table 1:** Bacterial strains and plasmids used in this study

Strain and plasmid	Relevant characteristic(s)	Source or reference
Strain		
*E. coli*		
DH5α	F^−^, *sup*E44, ΔlacU169 (φ80*lacZ*ΔM15), *hsd*R17, *rec*A1, *end*A1, *gyr*A96, *thi*-1, *rel*A1	Life technologies
GB05-red	An *E. coli* DH10B derive by deletion of *fhuA*, *ybcC* and *recET*, and insertion of the P_BAD_-*gbaA* cassette at the *ybcC* locus	[25]
*S. cellulosum*		
So0157-2	Epothilone producing strain	This lab
*M. xanthus*		
DK1622	Wild type	[36, 37]
DZ2	Wild type	[38]
SW504	*dif* mutant, contains a *difA* in frame deletion	[22, 23]
Plasmid		
pACYC184	*p15A* ori; Cm^r^	Novagen
pKK843	With an 843-bp epothione promoter *epop* inserted in front of the promoter-less cat gene (*epop*-*cat*); Cm^r^	[17]
pKK-aphII	With an *aphII* promoter fragment inserted in front of the promoter-less cat gene (*aphII*-*cat*); Cm^r^	[17]
pMiniHimar-lacZ	Himar1 transposon *(IR*-*Tpase*-*IR)*, *lacZ*; Km^r^	[13, 14]
pSWU30	Site specific integration vector with *Mx8 attB* integration site (*Mx8)*; Tet^r^	[27]
p15A-epop-cat	Ligating the *epop*-*cat* to the *p15A* ori; Cm^r^	This study
p15A-aph-cat	Ligating the *aphII*-*cat* to the *p15A* ori; Cm^r^	This study
pTp-epop	Ligating the *IR*-*Tpase*-*IR* to p15A-epop-cat; Cm^r^	This study
pTp-aph	Ligating the *IR*-*Tpase*-*IR* to p15A-aph-cat; Cm^r^	This study
pMx8-epop	Ligating *Mx8* to p15A-epop-cat; Cm^r^	This study
pMx8-aph	Ligating *Mx8* to p15A-aph-cat; Cm^r^	This study
Cosmid10	A plasmid from a Cosmid library of So0157-2 genome, containing foremost part of epothilone gene cluster (*epoA, epoP, epoB, epoC* and part of *epoD*); Amp^r^, Neo^r^	This study
Fosmid3B11	A plasmid from a fosmid library of So0157-2 genome, containing latter part of epothilone gene cluster (partial *epoC*, *epoD*, *epoE*, *epoF*); Cmr	This study
pSL1180	Cloning vector; Amp^r^	Pharmacia
pSL-UCD	Ligating the 2.0-kb *epoup* fragment, 2.2-kb *epoCov* fragment, and the 2.2-kb *epodown* fragment into pSL1180; Amp^r^	This study
pBJ113	Gene replacement vector with KG cassette; Km^r^	[28]
pGEM-Teasy	Cloning vector, Ampr	Promega
pGEM-galk	Ligating the 1.7-kb *galk* gene to pGEM-Teasy; Ampr	This study
pSL-ETG	Ligating the 3.2-kb TG cassette to pSL-UCD; Amp^r^, Tet^r^	This study
pCVD442	A suicide plasmid; Amp^r^	[29]
pSL-ETGS	Ligating the 1.8-kb *sacB* gene to pSL-ETG; Amp^r^, Tet^r^	This study
pSET152	Conjunction Vector; Apra^r^	This study
p15A-apra-cm	Ligating 1.7-kb *apra* fragment to p15A-aph-cat; Apra^r^, Cmr	This study
p15A-apra-cm-Tp	Ligating *IR*-*Tpase*-*IR* to p15A-apra-cm; Apra^r^, Cmr	This study
p15A-apra-cm-Mx8	Ligating *Mx8* to p15A-apra-cm; Apra^r^, Cmr	This study
p15A-recTp	Ligating the 11.5-kb MunI-SpeI fragment from pSL-ETGS to p15A-apra-cm-Tp; Apra^r^, Cmr, Tet^r^	This study
p15A-recMx8	Ligating the 11.5-kb MunI-SpeI fragment from pSL-ETGS to p15A-apra-cm-Mx8; Apra^r^, Cmr, Tet^r^	This study
p15A-TP-fos	Recombination between the linearized Fosmid3B11 and p15A-recTp; Apra^r^, Cmr, Tet^r^	This study
p15A-Mx8-fos	Recombination between the linearized Fosmid3B11 and p15A-recMx8; Apra^r^, Cmr, Tet^r^	This study
p15A-Tp-CF	Recombination between the linearized Cosmid10 and p15A-TP-fos; Apra^r^, Cmr	This study
p15A-Mx8-CF	Recombination between the linearized Cosmid10 and p15A-TP-fos; Apra^r^, Cmr	This study

### Construction of CAT reporter vectors

We amplified the 836-bp replicon *p15A* from pACYC184 using the primer pair of P15A. The chloramphenicol acetyl transferase (*cat*) gene was initiated by a promoter of either the epothilone operon promoter (*epop*) or the kanamycin promoter (*aphII*). These two primers are recognizable in *E. coli* and *M. xanthus* [10, 11, 17]. The 1.6-kb NdeI-KpnI fragment *epop*-*cat* was amplified from the pKK843 plasmid [17] using the CM-EPOP primer pair, while the 1.1-kb NdeI-KpnI fragment *aphII*-*cat* was amplified from pKK-aphII [17] using the CM-APH primer pair. The *epop*-*cat* and *aphII*-*cat* were ligated to the *p15A* replicon, generating the vectors of p15A-epop-cat and p15A-aph-cat, respectively. The 2.0-kb transposon element *IR*-*Tpase*-*IR* was amplified from plasmid pMiniHimar-*lacZ* [13] using the TPASE prime pair, while the 3.0-kb site-specific recombination element *Mx8* was generated from the template pSWU30 [27] using the MX8 primer pair. Finally, the plasmids pTp-epop (or pTp-aph) and pMx8-epop (or pMx8-aph) were constructed by ligating the *IR*-*Tpase*-*IR* and *Mx8* to p15A-epop-cat (or p15A-aph-cat), respectively, which had been digested with NdeI; and the DNA ends were blunted using the T4 DNA polymerase. The PCR primer pairs used in this study are summarized in Additional file 5: Table S3.

### Stitching and recombination of epothilone gene cluster

We constructed cosmid and fosmid libraries of *Sorangiumcellulosum* So0157-2 genome. Southern blotting hybridization with epothilone gene probes revealed clones containing the epothilone genes. The harbored epothilone genes fragments in the positive clones were determined by the end sequencing. We selected Cosmid10 and Fosmid3B11 for our construction. After completely sequencing, Cosmid10 contained a fragment of complete *epoA to epoC* and part of *epoD*, while Fosmid3B11 contained part of *epoC* till the downstream sequence of *epoF*.

To stitch the two epothilone-genes fragments in Cosmid10 and Fosmid3B11, transitional recombinant vectors p15A- recT and p15A-recM were constructed as follows. First, the 2.0-kb BglII-XbaI epoup fragment (upstream of *epoA*), 2.2-kbXbaI-NdeI epoCov fragment (overlapping the *epoC* sequence), and the 2.2-kb NotI-SpeI epodown fragment (downstream of epoF) were amplified from the genomic DNA of *S. cellulosum* So0157-2 using the primer pairs of UP, C-LAP and DOWN, respectively. The three PCR products, used as the homologous arms for recombination, were ligated serially into the corresponding restriction sites of pSL1180, generating pSL-UCD. Then, the 1.7-kb *galk* gene, containing XbaI and NheI sites at the 5′ end and XbaI site at the 3′ end, was amplified from pBJ113 [28] using the GALK primer pair, which was further ligated into the XbaI site of pGEM-Teasy vector, generating pGEM-galk. The 1.5-kb NheI *tet* fragment (tetracycline gene), amplified from pSWU30 using the TET primer pair, was ligated into the NheI site of the *galk* fragment in pGEM-galk. The 3.2-kb *tet*-*galk* fragment (TG cassette), used for positive and negative selection, was cleaved with Xba I, and ligated into the XbaI site of pSL-UCD, generating pSL-ETG. The plasmid pSL-ETG was cleaved with NdeI and ligated with the 1.8-kb Nde I *sacB* gene, which was amplified from pCVD442 [29] using the SACB primer pair, producing pSL-ETGS.

In another route, a 1.7-kb *apra* fragment (apramycin gene), amplified from pSET152 [30] using the APRA primer pair, was cloned into NdeI site of p15A-aph-cat to create p15A-apra-cm. Then, the PCR fragment *IR*-*Tpase*-*IR* or *Mx8* was ligated into p15A-apra-cm, which thereafter was cleaved at the 3′ end of the cat gene by ScaI, yielding the plasmid p15A-apra-cm-Tp and p15A-apra-cm-Mx8, respectively. Finally, the 11.5-kb MunI-SpeI fragment from pSL-ETGS with the blunt ends was cloned into the p15A-apra-cm-Tp, which had been cleaved with SnaBI with a blunt end, to create the purposed vector p15A- recTp. The plasmid was cloned into p15A-apra-cm-Mx8, which was further cleaved with KpnI, forming the blunt ends, to create the other purposed vector p15A-recMx8.

The vectors p15A-recTp and p15A-recMx8 was subsequently engineered for recombining with the epothilone gene cluster from Cosmid10 and Fosmid3B11. First, both Cosmid10 and Fosmid3B11 were cleaved with DraI, removing the irrelevant sequences and exposing the terminal sequences with epothilone genes, which contained the identical homologous arms epoup, epoCov and epodown. Then, the linearized Fosmid3B11 was electroporated into the *E. coli* strain GB05-red harboring p15A-recTp or p15A-recMx8 for the first round of recombination and the transformants with the objective recombinant p15A-TP-fos or p15A-Mx8-fos were selected on the LB plate containing Apra and Cm with extra 10% sucrose for negative selection. Similarly, in the second round the linearized Cosmid10 was electroporated into *E. coli* GB05-red harboring the plasmid from the first round and the transformants were selected on the LB plate containing extra 1% 2-deoxygalactose (DOG). Ultimately, the engineered recombinant plasmids p15A-Tp-CF and p15A-Mx8-CF containing the integrated epothilone gene cluster were constructed. All the above PCR products were amplified using the *pfu* DNA polymerase to ensure high fidelity and sequenced to confirm their identities. A diagrammatic sketch for the construction is provided in Figure 2.

### Electro-transformation of *M. xanthus*

The constructed expression plasmids were introduced into *M. xanthus* cells by electroporation. Briefly, *M. xanthus* cells from 50-mL overnight cultures (OD_600_ was about 0.6) were collected and washed thrice with ice-cold water for preparing competent cells. 1.5 mL of the culture was centrifuged and the precipitate was resuspended with 50 μL of ice-cold water in tube, mixed with 3 μg of DNA, and electroporated at a voltage of 1,250 V in a 2 mm cuvette using the Electroporator (Eppendorf, Germany). Then the cells were transferred into 2 mL CYE liquid medium in a 10-mL tube and incubated at 30°C with a rotate speed of 250 rpm. After 4–6 h of incubation, 0.1–0.5 mL of cells was added to 2 mL 0.5% soft agar and the mixture was spread on 1.5% CYE selection agar plates. Resistant colonies that appeared after 6 days of incubation were checked by colony PCR with the following primers: primer pairs of CM-APH and CM-EPOP were used to check the integration of *cat* gene; Epothilone-specific primers, EA, EP, EB, EC1, EC2, EE and EF, with a 51% coverage of the whole gene cluster, were designed to detect the regions located in ORFs *epoA,epoP*, *epoB*, *epoC*, *epoE* or *epoF*, respectively, to verify the integration of the whole biosynthetic gene cluster in the *M. xanthus* chromosome.

### Measurement of chloramphenicol acetyl transferase (CAT) activity in *M. xanthus*

To measure the chloramphenicol acetyl transferase (CAT) activity, the bacterial pellets were harvested by centrifugation at 8,000×*g* for 5 min after the cultures reached the mid-exponential phase (36 h of incubation; OD_600_ was about 3.0). The pellet was resuspended and washed twice with distilled water and then resuspended in 1 mL of lysis buffer. The crude bacterial extracts were prepared by sonication, and the supernatant was collected by centrifugation at 12,000×*g* for 2 min, and used for characterizing chloramphenicol acetyl transferase (cat) gene expression activity with the CAT ELISA kit, as previously described [17].

### Epothilone extraction and detection

*M. xanthus* strains containing the complete epothilone gene cluster were grown overnight in 100 mL CYE medium supplemented with Apra and Cm antibiotics. The cultures were inoculated at a ratio of 2:100 into CYE medium containing 2% of the XAD-16 resin for the absorption of epothilone products. After 7 days rotation at the 250 rpm speed and 30°C, the mixtures of cells and resin were harvested by centrifugation and extracted with two volume of methanol by shaking at room temperature overnight [31]. After centrifuged for 20 min, the supernatant was moved into a rotary evaporator to remove the solvent. The residue was further re-dissolved in 1 mL of methanol, and an aliquot of 20 μL of the sample was injected into a Finnigan HPLC system interfaced with a Finnigan MSQ classic quadrupole mass spectrometer (Thermo Finnigan, USA). The analysis was carried out on a Shim-pack MRC-ODS RP column (4.6 mm  ×  250 mm, 4.60 μm; Shimadzu, Japan) at a temperature of 28°C with a mobile phase of 60% methanol (HPLC grade) and 40% buffer (0.2% acetate acid/18 MΩ Millipore water) at a flow rate of 1.0  mL/min. The MS analysis was performed under the following conditions: ESI-positive, probe temperature of 450°C, cone voltage of 75  V, full scan mass range from 200 to 2,000  m/z at 2 Hz scan speed, and SIM scan at 494 [M + H]^+^ for epothilone A and 508 [M + H]^+^ for epothilone B [32]. Epothilones were identified by comparison to the retention time, 249  nm of UV spectra and the MS^2^ pattern of the authentic reference standard sample containing epothilones A and B, which were obtained from the culture of *S. cellulosum* So0157-2 as reported previously [31, 33]. The production levels of epothilones were averaged from three independent cultivations and extractions.

### Determination of the integration sites

A Genome Walking kit (TaKaRa, Japan) was used to verify the insertion sites of the gene integration. Three specific primers, Cm1, Cm2 and Cm3, designed in this work (Additional file 5: Table S3) were employed with the random primer AP1, which is provided by the kit, in three PCR rounds, to amplify a product containing partial cat region, the IR sequence of the transposon and the flanking unknown genome region of *M. xanthus*, respectively. Genomic DNA of the transformants with the epothilone gene cluster was extracted and used as the template in the first round PCR using the primer pair AP1/Cm1; the diluted product from the first PCR round was orderly used as the template for the second round, using the primer pair AP1/Cm2; and the product from the second round was diluted as the template for the third PCR round, using the AP1/Cm3 primer pair. The PCR systems and procedures were performed according to the kit protocol. The PCR products were detected by electrophoresis, and the single DNA bands with appropriate size from the third PCR round were withdrawn by the DNA Extraction kit (Promega, USA) and sequenced after subcloning to the pGEM-Teasy vector.

### Transcriptomics analysis

In this study, we exploit the ssRNA-seq method to identify the transcriptional template strands of *M. xanthus* DZ2 and three transformants ZE-5, ZE-9 and ZE-14 at a whole genome level using the Illumina-platform high throughput sequencing. First of all, the total RNA of the *M. xanthus* strains were extracted according to the protocol provided by the SV Total RNA isolation system kit (Promega, USA). Residual genomic DNA was removed by treatment with recombinant DNase I (RNase-free; Ambion, USA) according to the manufacturer’s instructions. The quality of the total RNA was verified by agarose gel electrophoresis, and the concentration was determined using a NanoDrop ND-1000 spectrophotometer (NanoDrop technologies, USA). Then, libraries were created by modifying the previously described dUTP second strand method [34]. We fragmented 200 ng of *M. xanthus* polyA^+^ RNA by heating at 98°C for 40 min in 0.2 mM sodium citrate, pH 6.4 (Ambion, USA). The fragmented RNA was concentrated to 5 μL, mixed with 3 μg random hexamers, incubated at 70°C for 10 min, and then cooled on ice. The RNA mixtures were further added with 4 μL of 5× first-strand buffer, 2 μL of 100 mM DTT, 1 μL of 10 mM dNTPs, 4 μg of actinomycin D (USB), 200 U SuperScript III, and 20 U SUPERase-In (Ambion, USA), incubated at room temperature for 10 min followed by 1 h at 55°C to synthesize the first-strand cDNA. First-strand cDNA was cleaned up by extraction twice with phenol: chloroform: isoamyl alcohol (25:24:1), followed by ethanol precipitation with 0.1 volumes 5 M ammonia acetate to remove dNTPs and re-suspension in 104 μL ddH_2_O. Second-strand cDNA was synthesized by adding 4 μL 5× first-strand buffer, 2 μL 100 mM DTT, 4 μL 10 mM dNTPs with dTTP replaced by dUTP (Sigma-Aldrich, USA), 30 μL 5× second strand buffer, 40 U Escherichia coli DNA polymerase, 10 U *E. coli* DNA ligase, 2 U E. coli RNase H and incubating at 16°C for 2 h. A paired-end library for Illumina sequencing was prepared according to the instructions provided with the following modifications. First, five times less adapter mixture was ligated to the cDNAs. Second, 1 U USER (New England Biolabs, USA) was incubated with 180- to 480-bp size-selected, adapter-ligated cDNA at 37°C for 15 min followed by 5 min at 95°C before PCR. Third, PCR was performed with Phusion High-Fidelity DNA Polymerase with GC buffer (New England Biolabs, USA) and 2 M betaine (Sigma, USA). Fourth, PCR primers were removed using 1.8× volume of AMPure PCR Purification kit (BeckmanCoulter Genomics, USA). Transcriptome sequencing was performed at the BGI Corporation. Reagents were all from Invitrogen (Carlsbad, USA) except as noted.

To assay the growth curves, *M. xanthus* colonies were first cultured overnight in CYE medium. Then, the cultures were transferred at a ratio of about 2:100 into 5 mL fresh CYE medium with a start OD_600_ of 0.15, and assayed of the OD_600_ values periodically.

The reference genome (*M. xanthus* DK1622) and the gene annotation was retrieved from GenBank (accession no. NC_008095.1). After removing the reads containing sequencing adapters and low-quality reads (reads containing Ns >10%), the remaining 90 bp clean reads with high quality were aligned with the reference genome using Bowtie software [35]. Then the RPKM method was used to normalize the transcript level, which was expressed as the number of reads per kilobase of exon region per million mapped reads (RPKM). Go annotation of the genes was performed using Blast2GO software and visualized by WEGO software.

## Additional files

Additional file 1:
**Figure S1.** Diagram for the construction of the CAT reporter gene vectors. The *epop* and *aphII* promoters were linked to *p15A* to generate p15-epop-cat and p15A-aph-cat, respectively. Then, the *IR-Tpase-IR* or *Mx8 attp* element was subcloned to each of the two plasmids above, respectively, to build the four final plasmids pTp-epoP, pMx8-epoP, pTp-aph and pMx8-aph, which were initiated by *aphII* and *epoP*, respectively. The plasmids were electroporated into *M. xanthus* to assay the promoter activities. Additional file 2:
**Table S1.** The insertion sites of the epothilone genes and the production of epothilones in different transposition recombinants. Additional file 3:
**Figure S2.** PCR amplification of the junction regions between the seven epothilone-modules. Genome templates for each PCR sample are as follows: 1, *M. xanthus* DZ2; 2, *M. xanthus* ZE5; 3, *M. xanthus* ZE9; 4, *M. xanthus* ZE14; 5, *S. cellulosum* So0157-2; 6, distilled water; M, DNA Marker DL2000 plus II. Additional file 4:
**Table S2.** Significantly up- and down-regulated *Myxococcus* genes in different recombinants. Additional file 5:
**Table S3.** Primers used in this study.
