# Supplementary material for: Allopatric integrations selectively change host transcriptomes, leading to varied expression efficiencies of exotic genes in Myxococcus xanthus
Source: Microb Cell Fact. 2015 Jul 22;14:105. doi: 10.1186/s12934-015-0294-5 (PMC4509775; doi:10.1186/s12934-015-0294-5)
Supplement: Additional file 1: — Figure S1. Diagram for the construction of the CAT reporter gene vectors. The epop and aphII promoters were linked to p15A to generate p15-epop-cat and p15A-aph-cat, respectively. Then, the IR-Tpase-IR or Mx8 attp element was subcloned to each of the two plasmids above, respectively, to build the four final plasmids pTp-epoP, pMx8-epoP, pTp-aph and pMx8-aph, which were initiated by aphII and epoP, respectively. The plasmids were electroporated into M. xanthus to assay the promoter activities. [file 12934_2015_294_MOESM1_ESM.pptx]

## Slide 1
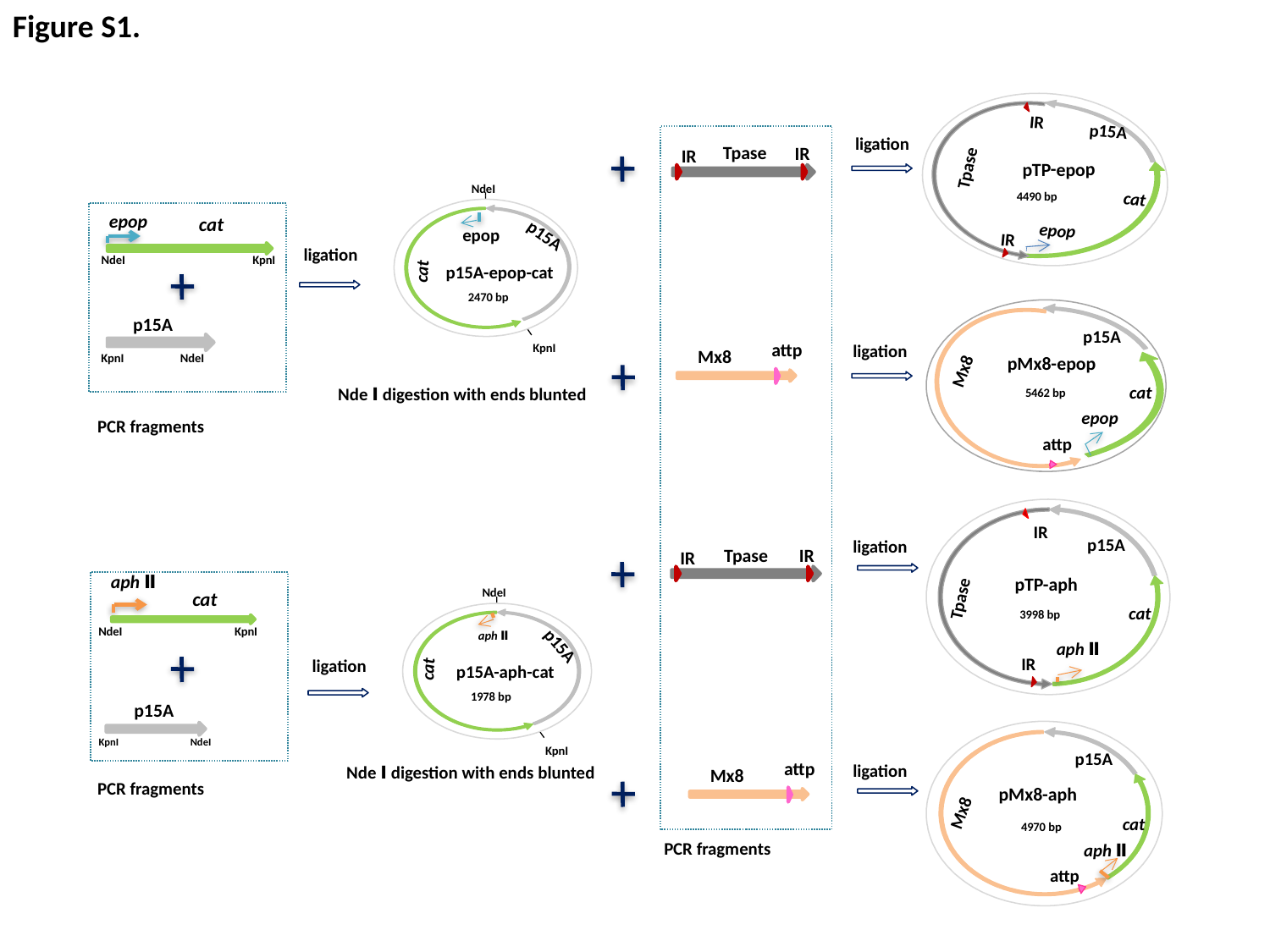

Figure S1.
IR
p15A
pTP-epop
Tpase
cat
4490 bp
IR
epop
ligation
Tpase
IR
IR
NdeI
p15A-epop-cat
2470 bp
KpnI
p15A
cat
epop
epop
cat
NdeI
KpnI
ligation
pMx8-epop
Mx8
5462 bp
attp
p15A
cat
epop
p15A
KpnI
NdeI
attp
Mx8
ligation
Nde Ⅰ digestion with ends blunted
PCR fragments
IR
p15A
pTP-aph
Tpase
cat
3998 bp
IR
aph Ⅱ
ligation
IR
Tpase
IR
aph Ⅱ
NdeI
KpnI
cat
NdeI
p15A-aph-cat
1978 bp
KpnI
p15A
aph Ⅱ
cat
ligation
p15A
KpnI
NdeI
pMx8-aph
Mx8
4970 bp
attp
p15A
cat
aph Ⅱ
attp
Mx8
ligation
Nde Ⅰ digestion with ends blunted
PCR fragments
PCR fragments
