# Supplementary material for: Allopatric integrations selectively change host transcriptomes, leading to varied expression efficiencies of exotic genes in Myxococcus xanthus
Source: Microb Cell Fact. 2015 Jul 22;14:105. doi: 10.1186/s12934-015-0294-5 (PMC4509775; doi:10.1186/s12934-015-0294-5)
Supplement: Additional file 2: — Table S1. The insertion sites of the epothilone genes and the production of epothilones in different transposition recombinants. [file 12934_2015_294_MOESM2_ESM.docx]

**Table S1.** **The insertion sites of the epothilone genes and the production of epothilones in different transposition recombinants**

| **Recombinants** | **Insertion site** | **Insertion Gene ID** | **epothiloneA yield (mg/L)** | **epothiloneB yield (mg/L)** | **Expressions of inserted genes in DZ2 (reads)** |
| --- | --- | --- | --- | --- | --- |
| KE-10 | 96846 | MXAN_0084 | 0.0398 | 0.204 | 23 |
| KE-20 | 96846 | MXAN_0084 | 0.0419 | 0.208 | 23 |
| SE-4 | 300656 | MXAN_0252 | 0.0084 | 0.043 | 42 |
| KE-21 | 330860 | MXAN_0277 | 0.135 | 0.664 | 213 |
| ZE-6 | 376169 | MXAN_0318 | 0.0924 | 0.461 | 104 |
| KE-2 | 522398 | MXAN_0458 | 0.0867 | 0.448 | 18 |
| ZE-8 | 700251 | MXAN_0605 | 0.0656 | 0.342 | 22 |
| KE-4 | 1411958 | MXAN_1204 | 0.0723 | 0.358 | 9 |
| ZE-7 | 1754653 | MXAN_1493 | 0.0956 | 0.473 | 26 |
| KE-6 | 1789724 | MXAN_1527 | 0.0622 | 0.318 | 15 |
| KE-30 | 1966726 | MXAN_1665 | 0.053 | 0.265 | 0 |
| KE-18 | 2165929 | MXAN_1840 | 0.0746 | 0.369 | 211 |
| KE-22 | 2165929 | MXAN_1840 | 0.0592 | 0.308 | 211 |
| KE-16 | 2165930 | MXAN_1840 | 0.0991 | 0.564 | 211 |
| ZE-10 | 2201002 | MXAN_1878 | 0.104 | 0.523 | 9 |
| ZE-12 | 2201002 | MXAN_1878 | 0.124 | 0.652 | 9 |
| KE-12 | 2550312 | MXAN_2209 | 0.0621 | 0.321 | 440 |
| KE-7 | 3390195 | MXAN_2898 | 0.0644 | 0.339 | 13 |
| KE-3 | 4136019 | MXAN_3552 | 0.102 | 0.511 | 127 |
| KE-27 | 4197364 | MXAN_3600 | 0.0467 | 0.236 | 106 |
| KE-15 | 4391011 | MXAN_3680 | 0.0757 | 0.389 | 22 |
| KE-17 | 4654434 | MXAN_3875 | 0.188 | 0.938 | 12 |
| KE-28 | 5072631 | MXAN_4130 | 0.0501 | 0.253 | 21 |
| ZE-13 | 5421017 | MXAN_4402 | 0.109 | 0.570 | 11 |
| **ZE-14** | 5421017 | MXAN_4402 | 0.128 | 0.664 | 11 |
| ZE-11 | 5549831 | MXAN_4482 | 0.143 | 0.724 | 19 |
| KE-9 | 5593331 | MXAN_4513 | 0.0431 | 0.216 | 6 |
| KE-26 | 5693439 | MXAN_4561 | 0.0678 | 0.334 | 43 |
| ZE-1 | 5774803 | MXAN_4601 | 0.152 | 0.745 | 46 |
| ZE-3 | 5797491 | MXAN_4619 | 0.0962 | 0.479 | 7 |
| **ZE-9** | 6262586 | MXAN_5011 | 0.208 | 1.049 | 15 |
| SE-1 | 6573297 | MXAN_5274 | 0.00481 | 0.025 | 6 |
| KE-19 | 7177554 | MXAN_5798 | 0.10137 | 0.507 | 89 |
| KE-24 | 7177554 | MXAN_5798 | 0.0897 | 0.453 | 89 |
| KE-25 | 7177554 | MXAN_5798 | 0.0829 | 0.414 | 89 |
| KE-5 | 7187383 | MXAN_5801 | 0.0448 | 0.229 | 45 |
| KE-11 | 7397507 | MXAN_5974 | 0.0431 | 0.215 | 89 |
| ZE-2 | 7697516 | MXAN_6233 | 0.111 | 0.505 | 1354 |
| SE-3 | 8627489 | MXAN_7050 | 0.0129 | 0.073 | 28 |
| SE-2 | 8660910 | MXAN_7087 | 0.00548 | 0.027 | 75 |
| KE-1 | 8706510 | MXAN_7132 | 0.0468 | 0.229 | 76 |
| KE-23 | 8859779 | MXAN_7258 | 0.0612 | 0.304 | 292 |
| KE-13 | 8910605 | MXAN_7294 | 0.132 | 0.711 | 0 |
| **ZE-5** | 8940497 | MXAN_7320 | 0.0449 | 0.222 | 26 |
| KE-8 | 8970540 | MXAN_7350 | 0.0691 | 0.346 | 223 |
| KE-14 | 8970540 | MXAN_7350 | 0.0807 | 0.403 | 223 |
| ZE-4 | 9086534 | MXAN_7460 | 0.0987 | 0.505 | 35 |
| KE-29 | 9098141 | MXAN_7469 | 0.0773 | 0.392 | 71 |
