# Supplementary figures and images for: Allopatric integrations selectively change host transcriptomes, leading to varied expression efficiencies of exotic genes in Myxococcus xanthus
Source: Microb Cell Fact. 2015 Jul 22;14:105. doi: 10.1186/s12934-015-0294-5 (PMC4509775; doi:10.1186/s12934-015-0294-5)

## Slide 1
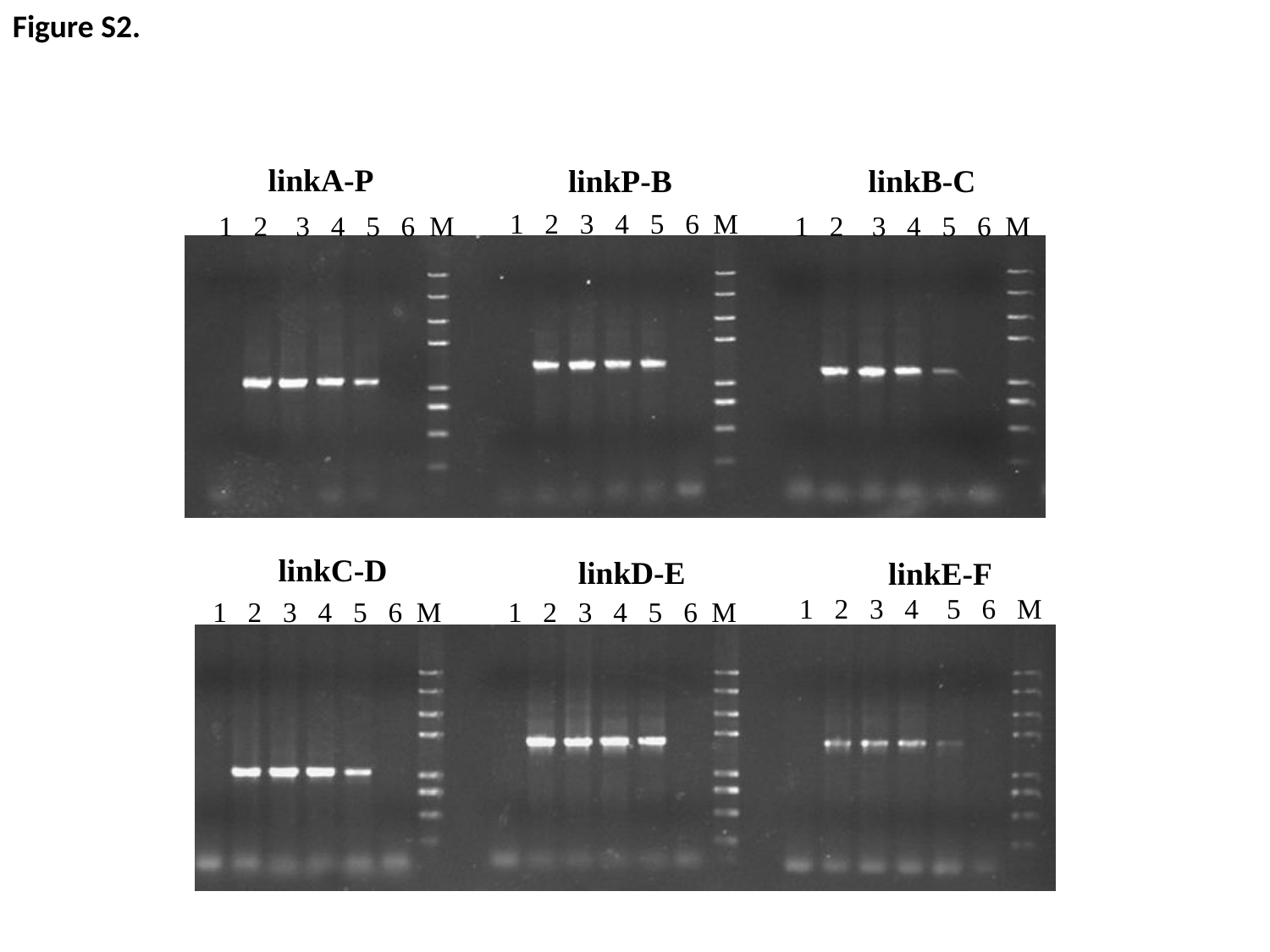

Figure S2.
linkA-P
linkP-B
linkB-C
1 2 3 4 5 6 M
1 2 3 4 5 6 M
1 2 3 4 5 6 M
linkC-D
linkD-E
linkE-F
1 2 3 4 5 6 M
1 2 3 4 5 6 M
1 2 3 4 5 6 M

Supplement: Additional file 3: — Figure S2. PCR amplification of the junction regions between the seven epothilone-modules. Genome templates for each PCR sample are as follows: 1, M. xanthus DZ2; 2, M. xanthus ZE5; 3, M. xanthus ZE9; 4, M. xanthus ZE14; 5, S. cellulosum So0157-2; 6, distilled water; M, DNA Marker DL2000 plus II. [file 12934_2015_294_MOESM3_ESM.pptx]
