# Supplementary material for: Allopatric integrations selectively change host transcriptomes, leading to varied expression efficiencies of exotic genes in Myxococcus xanthus
Source: Microb Cell Fact. 2015 Jul 22;14:105. doi: 10.1186/s12934-015-0294-5 (PMC4509775; doi:10.1186/s12934-015-0294-5)
Supplement: Additional file 4: — Table S2. Significantly up- and down-regulated Myxococcus genes in different recombinants. [file 12934_2015_294_MOESM4_ESM.docx]

**Table S2. Significantly up- and down-regulated *Myxococcus* genes in different recombinants***

| **Gene** | **ZE-5** | **ZE-9** | **ZE-14** | **Predicted Function** |
| --- | --- | --- | --- | --- |
| MXAN_4366 | **up6.4*** | NS | NS | hypothetical protein |
| MXAN_0807 | **up6.2** | NS | NS | hypothetical protein |
| MXAN_4368 | **up5.3** | NS | NS | hypothetical protein |
| MXAN_4525 | **up4.4** | NS | NS | non-ribosomal peptide synthase MxaA |
| MXAN_7320 | **up4.3** | NS | NS | PheA/TfdB family FAD-binding monooxygenase |
| **MXAN_4110** | **up4.2** | NS | NS | LysR family transcriptional regulator |
| MXAN_1852 | **up3.9** | NS | NS | phage tail sheath protein |
| MXAN_6104 | **up3.8** | NS | NS | hypothetical protein |
| **MXAN_6484** | **up3.6** | NS | NS | ExbD/TolR family transport energizing protein |
| **MXAN_0882** | **up3.6** | NS | NS | serine/threonine protein kinase |
| MXAN_4369 | **up3.5** | NS | NS | hypothetical protein |
| MXAN_5134 | **up3.1** | NS | NS | MutS domain-containing protein |
| **MXAN_1853** | **up3.0** | NS | NS | heme-binding protein |
| MXAN_0876 | **up2.6** | NS | NS | group 2 family glycosyl transferase |
| MXAN_5278 | **up2.5** | NS | NS | hypothetical protein |
| **MXAN_0453** | **up2.4** | NS | NS | general stress protein 26 |
| **MXAN_0578** | **up2.4** | NS | NS | TonB family protein |
| **MXAN_3650** | **up2.3** | NS | NS | permease |
| **MXAN_3547** | **up2.2** | NS | NS | 16S rRNA-processing protein RimM |
| MXAN_0648 | **down4.7** | NS | NS | hypothetical protein |
| **MXAN_0681** | **down4.7** | NS | NS | ECF subfamily RNA polymerase sigma factor |
| MXAN_2421 | **down3.1** | NS | NS | hypothetical protein |
| MXAN_3174 | **down3.1** | NS | NS | hypothetical protein |
| MXAN_6413 | **down8.6** | NS | NS | DNA-binding response regulator PhoP3 |
| MXAN_1576 | **up4.3** | **up6.9** | **up7.2** | major facilitator family transporter |
| MXAN_1093 | **up2.9** | **up3.7** | **up3.2** | DNA-binding response regulator |
| MXAN_7163 | **up4.1** | **up6.5** | **up6.9** | bis(5'-nucleosyl)-tetraphosphatase, symmetrical |
| MXAN_7372 | **down7.3** | **down6.6** | **down7.8** | hypothetical protein |
| MXAN_4372 | **down6.1** | **down4.3** | **down7.7** | DNA-binding protein |
| MXAN_5126 | NS | **up3.4** | **up4.2** | hypothetical protein |
| MXAN_5530 | NS | **up2.9** | **up3.0** | hypothetical protein |
| MXAN_3967 | NS | **up2.5** | **up3.2** | hypothetical protein |
| MXAN_4480 | NS | **down2.1** | **down3.0** | DNA-binding protein |
| MXAN_4324 | NS | **down2.5** | **down2.3** | hypothetical protein |
| MXAN_0509 | NS | **up3.1** | NS | hypothetical protein |
| MXAN_0259 | NS | **down1.7** | NS | response regulator |
| MXAN_1511 | NS | **down1.7** | NS | hypothetical protein |
| MXAN_0977 | NS | **down1.7** | NS | di-heme cytochrome-c peroxidase |
| MXAN_0979 | NS | **down2.2** | NS | copper-translocating P-type ATPase |
| MXAN_7463 | NS | **down4.0** | NS | ornithine cyclodeaminase |
| MXAN_3382 | NS | **down3.3** | NS | cation efflux family protein |
| MXAN_5125 | NS | NS | **up2.5** | transcriptional regulator MrpC |
| MXAN_2369 | NS | NS | **down1.8** | 3'(2'),5'-bisphosphate nucleotidase |
| MXAN_0200 | NS | NS | **down2.6** | hypothetical protein |

* The up- or down-regulated genes were compared with DZ2. The number, in Log (mutant/DZ2), with higher than 2 is considered to be those differentially expressed. “NS” (no significant change) “NS” refers to either the q value of the gene expression quantity is more than 0.05 or the log value is less than 1.
