# Supplementary material for: Allopatric integrations selectively change host transcriptomes, leading to varied expression efficiencies of exotic genes in Myxococcus xanthus
Source: Microb Cell Fact. 2015 Jul 22;14:105. doi: 10.1186/s12934-015-0294-5 (PMC4509775; doi:10.1186/s12934-015-0294-5)
Supplement: Additional file 5: — Table S3. Primers used in this study. [file 12934_2015_294_MOESM5_ESM.docx]

**Table S3. Primers used in this study**

| **Name** | **Direction** | **Sequence*** | **Site** |
| --- | --- | --- | --- |
| P15A | Forward | 5’- ggGGTACCATCTGATTAATAAGATGATCTTC-3’ | Kpn I |
|  | Reverse | 5’- ggaattcCATATGCGCTAGCGGAGTGTATA -3’ | Nde I : |
| CM-EPOP | Forward | 5’-ggaattcCATATGGCCTTGGTCATGTGGTGTTC-3’ | Nde I |
|  | Reverse | 5’-ggGGTACCCACTTATTCAGGCGTAGCA-3’ | Kpn I |
| CM-APH | Forward | 5’-ggaattcCATATGGCTTCACGCTGCCGCAAGCAC-3’ | Nde I |
|  | Reverse | 5’-ggGGTACCCACTTATTCAGGCGTAGCA-3’ | Kpn I |
| TPASE | Forward | 5’CGCCTTCTTGACGAGTTC-3’ | none |
|  | Reverse | 5’ATTCCGGAGTATACGTAGCC-3’ | none |
| MX8 | Forward | 5’GATCTTCACCTGGGGATGGAG-3’ | none |
|  | Reverse | 5’GGATGCGGTGGACCATGAGC-3’ | none |
| APRA | Forward | 5’-ggaattcCATATGGTTCATGTGCAGCTCCATCAG-3’ | NdeI |
|  | Reverse | 5’-ggaattcCATATG GAAGGGCAGCCCACCTATC-3’ | NdeI |
| UP | Forward | 5’-gaAGATCTCGGACTTGACGACCTTGCTG-3’ | BglII |
|  | Reverse | 5’-gcTCTAGAGCGACAACATCAGCGATACCC-3’ | XbaI |
| C-LAP | Forward | 5’-gcTCTAGAGAGCTACTGGGCGGACAACG-3’ | XbaI |
|  | Reverse | 5’-ggaattcCATATGGTGGGTGCCGAAACTGAAGG-3’ | NdeI |
| DOWN | Forward | 5’-ataagaatGCGGCCGCGCTGGTGAAGCGAACGAGACG-3’ | Not I |
|  | Reverse | 5’-ggACTAGTTGGAGGAGCGAAGGCTGACG-3’ | Spe I |
| APRA | Forward | 5’-ggaattcCATATGGTTCATGTGCAGCTCCATCAG-3’ | NdeI |
|  | Reverse | 5’-ggaattcCATATG GAAGGGCAGCCCACCTATC-3’ | NdeI |
| GALK | Forward | 5’-gcTCTAGA GCTAGC CGAAATGACCGACCAAGC-3’ | XbaI -NheI |
|  | Reverse | 5’-gcTCTAGA ATACACTCCGCTATCGCTACG-3’ | XbaI |
| TET | Forward | 5’-ctaGCTAGC TCCGCCTCCATCCAGTC-3’ | NheI |
|  | Reverse | 5’-ctaGCTAGC CATTCACAGTTCTCCGCA AG-3’ | NheI |
| SACB | Forward | 5’-ggaattcCATATGGAGTCTAATAGAATGAGGTCGA-3’ | NdeI |
|  | Reverse | 5’-ggaattcCATATGAAGTCATCGGGCATTATCT-3’ | NdeI |
| EA | Forward | 5’-GTGCTAGTGATCTGCCTTACGTTGTG-3’ | none |
|  | Reverse | 5’-CATAGGGCAATGATTTCCCAGTCT-3’ | none |
| EP | Forward | 5’-CCTATGACGATCAATCAGCTTC-3’ | none |
|  | Reverse | 5’-GATGAATCCCAGGGTGAGGC-3’ | none |
| EB | Forward | 5’-CGCAAGTCCGCCTGCCTCA-3’ | none |
|  | Reverse | 5’-AGCGGATTCTGCTGTGCC-3’ | none |
| EC1 | Forward | 5’-ACACGAGGCATCGGGAATCTG-3’ | none |
|  | Reverse | 5’-TCGGTCTCCTGCGACAATAGC-3’ | none |
| EC2 | Forward | 5’-GGGGATGAAAGCCTGGAGAC-3’ | none |
|  | Reverse | 5’-GCCGAGACGAGCAATGAAGC-3’ | none |
| EE | Forward | 5’-TGGTGAGTAAGGGACCGAGG-3’ | none |
|  | Reverse | 5’-GGAGGCTGCCATCATTTT-3’ | none |
| EF | Forward | 5’-ATGACGCAGGAGCAAGCGAATC-3’ | none |
|  | Reverse | 5’-GCGAGTTATCCAGCTTTGGAGGG-3’ | none |
| Cm1 | Forward | 5’-CACATTCTTGCCCGCCTGAT-3’ | none |
| Cm2 | Forward | 5’-GGCAATGAAAGACGGTGAGC-3’ | none |
| Cm3 | Forward | 5’-TGCCGTCTGTGATGGCTTCC-3’ | none |

*the restriction enzyme sites are underlined.
